# *Salmonella* nomenclature in the genomic era: a time for change

**DOI:** 10.1038/s41598-021-86243-w

**Published:** 2021-04-05

**Authors:** Marie A. Chattaway, Gemma C. Langridge, John Wain

**Affiliations:** 1 Gastrointestinal Bacteria Reference Unit, Salmonella Reference Service, Public Health England, London, NW9 5EQ UK; 2 Quadram Institute Bioscience, Norwich Research Park, Norwich, NR4 7UQ UK; 3 Norwich Medical School, University of East Anglia, Norwich, NR4 7TJ UK

**Keywords:** Evolution, Genetics, Microbiology, Molecular biology, Diseases

## Abstract

*Salmonella enterica* nomenclature has evolved over the past one hundred years into a highly sophisticated naming convention based on the recognition of antigens by specific antibodies. This serotyping scheme has led to the definition of over 2500 serovars which are well understood, have standing in nomenclature and, for the majority, biological relevance. Therefore, it is highly desirable for any change in naming convention to maintain backwards compatibility with the information linked to these serovars. The routine use of whole genome sequencing and the well-established link between sequence types and serovars presents an opportunity to update the scheme by incorporating the phylogenetically relevant sequence data whilst preserving the best of serotyping nomenclature. Advantages include: overcoming the variability in antibody preparations; removing the need to use laboratory animals and implementing a truly universal system. However, the issue of trying to reproduce the phenotyping gold standard needs to be relaxed if we are to fully embrace the genomic era. We have used whole genome sequence data from over 46,000 isolates of *Salmonella enterica* subspecies *enterica* to define clusters in two stages: Multi Locus Sequence Typing followed by antigen prediction. Sequence type—serotype discrepancies were resolved using core SNP clustering to determine the phylogenetic groups and this was confirmed by overlaying the antigenic prediction onto the core SNP clusters and testing the separation of clusters using cgMLST Hierarchical Clustering. This allowed us to define any major antigenic clusters within an ST—here called the MAC type and written as ST-serovar. Using this method, 99.96% of *Salmonella* isolates reported in the UK were assigned a MAC type and linked to a serovar name taken from the Kauffmann and White scheme. We propose a change for reporting of *Salmonella enterica* sub-types using the ST followed by serovar.

## Introduction

We identify and name pathogenic organisms because in many cases this informs clinical and public health management of the diseases they cause. For *Salmonella,* the second most common cause of bacterial food poisoning, specific antibodies are used which recognise cell wall (O) and phase 1 and phase 2 flagella (H) antigens. The combination of O:H1:H2 is known as the antigenic formula and each unique combination is given a serovar (or serotype) name. Clinically the most critical differentiation for *Salmonella* is between the enteric fever-causing typhoidal serovars and the non-typhoidal (NTS) serovars that most commonly cause gastroenteritis. Enteric fever requires antibiotic therapy and if a typhoidal serovar: *Salmonella enterica* Typhi or Paratyphi, is identified then contact tracing is considered. For infection with NTS, specific therapy is not required in the immunocompetent host but because of the impact on society, outbreak investigations are often initiated where there is an exceedance in the population of a clonal strain. In the UK, if an isolate is identified as *Salmonella* then under the Public Health (Control of Disease) Act 1984 this must, by law, be notified to Public Health England and so typing carries both a legal and a public health responsibility. The local Health Protection Team is informed and after confirmation and typing from the reference laboratory the case is included in the national databases for infection surveillance; outbreak investigations are initiated if appropriate.

*Salmonella* is currently classified into two species, *Salmonella bongori* (originally classified as *Salmonella enterica* subspecies V), rarely associated with human infection, and *Salmonella enterica*. *S. enterica* is a diverse species which infects and colonises many animals including humans. Historically *S. enterica* classification has been based on biochemistry, to define 6 subspecies (I *enterica*, II *salamae*, IIIa *arizonae*, IIIb *diarizonae*, IV *hountenae,* VI *indica*)^1^. Most human infections involve serovars within subspecies I^2^, which are named according to the Kauffmann-White scheme^1,3^. The first published Kauffmann-White Scheme (1934) described 44 serovars^4^ and the latest (2007) contains over 2500^1^. The approach can be inconsistent, particularly in cases where sub-typing of serovars by the use of biochemical properties is necessary; these are termed biovars (or biotypes). For example, isolates with the antigenic formula 4,5,12:b:1,2, are sub-divided by the ability to utilise d-tartrate into *S.* Paratyphi B (d-tartrate − ve) and *S.* Paratyphi B *var* Java (d-tartrate + ve). However, the serovar *S*. Java was withdrawn from the Kauffman-White 9th edition^1^. Human infection with these biovars results in very different clinical outcomes and laboratories in the UK require different containment levels; clear differentiation would therefore be very useful. Another example of *Salmonella* nomenclature issues using the gold standard is *Salmonella enterica* subspecies VII^5,6^ which has not been formally recognised because biochemical analysis misidentifies strains^1^. These examples raise the question “should we continue to define *Salmonella* phenotypically for formal recognition?”.

The introduction of sequence-based methods such as multi-locus sequence typing (MLST) has allowed reanalysis of the *S. enterica* population structure with phylogenetically relevant methods. Isolates that possess seven identical alleles, at the DNA sequence level, of conserved housekeeping genes are assigned to the same sequence type (ST). Sequence types cluster into groups of single locus variants where each individual ST shares 6/7 alleles with at least one other ST in the group; these clonal complexes^7^ are known as eBURST groups (eBGs)^8^ and relate very closely to serovars^9^. In 2014, PHE implemented whole genome sequencing (WGS) and validated the naming of serovars by referral to ST^10^. Another approach is to reproduce the entire Kauffmann-White scheme using the genetic sequence of every antigen encoding gene to predict the antigen encoded—genoserotyping. Programmes such as SeqSero^11^ have been developed with this aim in mind and are very successful; 98% concordance with serotyping reported from routine use on 520 isolates (20 serotypes)^12^ but the genetic basis for some antigens, particularly the cell wall (O) antigens remains elusive. The combination of the two sequence based approaches, phylogenetic methods with genoserotyping, for example the *Salmonella *in Silico Typing Resource (SISTR)^13^, reports 94.6% concordance on a dataset comprised of 4188 *Salmonella* genomes. However, comparison with the gold standard of the Kauffmann-White scheme is not perfect and remains a controversial topic^14^. Where WGS had been implemented^10^ the use of genoserotyping has shown 89% concordance with the gold standard: of 17,899 confirmed *Salmonella* laboratory results reported between April 2016 and March 2018, the serovar of 15,945 (89.1%) *Salmonella* were reported by using sequence type (ST) combined with SeqSero. However, for complete resolution 3,678 (20.6%) isolates required antibody-based serotyping using antibodies raised in rabbits. The main reason for antibody-based serotyping was discrepancy between the sequence predicted serovar and that reported by the sending laboratory with a smaller proportion attributed to novel *Salmonella* and differentiation of complex *Salmonella* groups^14^.

*Salmonella* typing is in transition, the Kauffmann-White Scheme has not been updated since 2007, and there is not yet agreement on what should replace it—genomic approaches are generally considered to be the most promising but consensus is needed to ensure a standardisation of approach. One of the main issues is with isolates for which the link between serovar and DNA sequence data is not clear. These problematic isolates fall into three groups: (1) unknown genetics of antigen production—genoserotyping has not been defined; (2) lack of concordance with gold standard—commonly serotyping differentiates two isolates which have the same ST; and (3) novel STs—new STs which have not been formally approved. If we continue to follow the formally recognised gold standard then serotyping with antibodies raised in rabbits will remain necessary for the foreseeable future^14^. The solution may be with international groups such as PulseNet International, a global network dedicated to laboratory-based surveillance for food-borne diseases which is the most widely accepted process for reviewing *Salmonella* typing. Their vision is the implementation of WGS surveillance^15^ however, as the focus of PulseNet is to reach a consensus for the definition of strains at the SNP and allele level for outbreak detection, the naming of *Salmonella* isolates at the serovar level remains in flux.

In this publication we look at the practical issue of naming *Salmonella* at the serovar level. We present analysis of the sequence data generated at Public Health England from all *Salmonella enterica* isolates referred from England and Wales to the *Salmonella* Reference Unit over 5 years; we focus on the problematic isolates and propose a method for naming all *Salmonella* isolates using WGS data—our aim is to remove the need for antibody based serotyping.

## Methods

*Salmonella enterica* subspecies *enterica* sequenced isolates^14^ reported between January 2014 and 21st June 2019 selected for this study included 46,268 strains. Data was analysed as follows:

### Serovar inference using MLST

Analysis was undertaken on the 46,268 strains to understand the number of isolates in which ST alone could be used for identification without the need for any further testing.

### Assigning isolates to novel STs

Since routine implementation in 2015, sequences that did not map to any known sequence type (i.e. novel ST) were assigned a new sequence type via PubMLST (https://pubmlst.org/Salmonella/) and validated by antibody-based testing. In brief, antibody-based testing was undertaken using in house sera antibodies against the isolate antigen using slide agglutination, microtitre dilution and Craigie motility agar methods according to the Kauffmann-White scheme^1,3^. At the time of this study, antibody-based serotyping was performed on three independent isolates before an ST was validated and then used to define a serovar^14^. Novel STs were also compared against SeqSero^11^ for identification and unresolved serovars were then assessed using predicted ST serovars in Enterobase as previously described^16^.

### Defining major antigen clusters (MACs) within problematic groups

The groups that could not be designated a serovar based on ST and analysis of antigen encoding genes were defined as being problematic. Usually this was because one ST contained more than one serovar, or the serovar prediction programmes were not able to assign a serovar. A random subset of strains from each group also had antibody-based testing as described above^1,3^. Problematic groups were analysed further to determine if the different serovars with the same ST came from distinct phylogenetic groups or were in fact from a single population—we term this the MAC type which was achieved as follows.

#### Somatic antigen clusters

At the time of writing, the available serovar prediction programmes were not able to resolve all O antigens from the genome sequence. Isolates from STs containing multiple serovars (defined by serological based typing of O antigens) were tested for phylogenetic separation using core genome SNP clustering.

#### Flagella clusters

We defined H antigens using serovar prediction programmes and reproduced the names in the Kauffman-White scheme for most of the problematic STs using SeqSero—we checked three for concordance with antibody-based serotyping. The necessity of using *fliC* sequences as a differentiating factor within a ST has been questioned^9^, therefore variation at the genomic level for serovars within a single ST that differed only by an H antigen was investigated using core genome SNP clustering.

#### Core genome SNP clustering

All data were analysed in Galaxy^17^. Raw Illumina sequence data (previously generated by PHE) was downloaded from the NBCI sequence read archive (SRA, https://www.ncbi.nlm.nih.gov/sra). Strains with associated laboratory serovar identifications were assembled using SPAdes^18^ (v3.12.0 + galaxy1) with default parameters and assessed with QUAST^19^ (v5.0.2). Per group, a high quality reference was selected that had the fewest contigs > 1000 bp and had the largest single contig. Reference assemblies were reduced to contigs > 1000 bp using seqtk_seq^20^ (v1.3.3) with the -L 1000 flag. All sequenced isolates per group were compared to the reference using snippy^21^ (v3.2) and snippy-core was used to generate a core SNP alignment. Maximum likelihood phylogenetic trees were generated using IQ-TREE^22^ (Galaxy version 1.6.12) and visualised with associated metadata in iTOL^23^. Differences in *fliC* and *fljB* nucleotide sequences were assessed by sequence comparison in Seaview^24^ (v4.7) after annotation of the reference sequence using prokka^25^ (Galaxy version 1.13). As ST226 (Carrau/Gatow) only comprised 9 isolates in the PHE sequences, this group was supplemented with 129 isolates obtained from Enterobase with the same ST and whole genome sequence available for download. Isolates assigned serovar Bredeney came from either ST241 or ST897. To assess whether these truly represented different phylogenetic groups, a maximum likelihood tree containing all isolates from ST241 and ST897 was generated and visualised as before.

#### Hierarchical clustering

Hierarchical Clustering of the groups was assessed using tools in Enterobase, an open access public database, as previously described^26^ to define the number of alleles (3002 cgMLST genes) at which the MAC types differentiate. This enables readers to differentiate the MAC types who may not have access to bespoke phylogenetic methods.

Major Antigenic Cluster (MAC) types were designated to both serovars if serovars were genetically distinct and differed by a minimum 100 alleles using cgMLST Hierarchical Clustering, otherwise the most common serovar was assigned as the MAC type^16,26^.

## Results

DNA sequence data was available from 46,268 subspecies I *Salmonella* isolates sequenced at PHE over the period January 2014 to June 2019 comprising 550 named serovars and 1704 STs. In this study, a total of 11,605/46,268 (25%) isolates had antibody-based testing undertaken. Using traditional serological techniques during this time period, there were 970 (2.1%) isolates reported as unnamed serovars. Using a genomic approach and utilising the ST to associate sequence to serovar, the naming of 43,657 (94.37%) isolates was possible, leaving 2601 (5.63%) requiring further analysis. These isolates fell into two main categories, 1774 (3.85%) isolates were associated with ‘problematic groups’ and 827 (1.78%) isolates were novel STs. SeqSero could differentiate 1,607 isolates from problematic groups in accordance with MAC typing and 806 isolates with novel STs. This left a total of just 172 isolates (0.37%) from problematic groups that couldn’t be fully resolved using ST and SeqSero according to the Kaufmann and White Scheme (see Supplementary. Fig S1), further details described in below.

### Problematic groups (1774 isolates)

Each problematic group was investigated (Table 1) and the differences in the somatic (O) antigen or flagella (H) antigen were recorded. Clustering on core SNP similarity was performed and the serovar name was overlaid to visualise the distribution of serovars on the SNP tree—where the serovars clustered independently, a major antigen cluster or MAC type was defined within the ST. See Fig. 1A,B for examples and Supplementary Figs. S2–S14 for details.

**Table 1:** Proposed reporting guidelines for *Salmonella* using genome sequence data to define the major antigenic clusters (MACs).

ST	Serotyping^a^	Differential antigen^b^	Phylogeny grouping	No. Isolates	%	Proposed MAC type/name^c^	Main hierarchical clustering level to differentiate	Figure
20	Brandenburg	H: l,v	Distinct	24	0.05	ST20-Brandenburg	HC400_11346	Figure 1A
20	Sandiego	H1: e,h	Distinct	26	0.06	ST20-Sandiego	HC400_2255	Figure 1A
1985	Bahati or Durham	O:22 or O:23	Mixed	66	0.14	ST1985-Durham	N/A—interspersed	Figure 1B
49	Saintpaul	H: e,h	Distinct	75	0.16	ST49-Saintpaul	HC200_8	Figure S2
49	Haifa	H: z10	Distinct	130	0.28	ST49-Haifa	HC200_1433	Figure S2
582	Kottbus	H: e,h	Distinct	42	0.09	ST582-Kottbus	HC900_7054	Figure S3
582	Chailey	H: z23	Distinct	6	0.01	ST582-Chailey	HC900_17	Figure S3
22	Braenderup	H2: e,n, z15	Distinct	607	1.31	ST22-Braenderup	HC100_185	Figure S4
22	Larochelle	H2: 1,2	Distinct	19	0.04	ST22-Larochelle	HC100_1136, 25669, 2664, 28707	Figure S4
241	Bredeney	H: l,v	Distinct	45	0.10	ST241-Bredeney	HC200_2494, HC200_1335	Figure S5, S14
241	Schwarzengrund	H: d	Distinct	12	0.03	ST241-Schwarzengrund	HC200_17392	Figure S5
897	Bredeney	H2: 1,7	Distinct	8	0.02	ST897-Bredeney	HC400_31544	Figures S6, S14
897	Kimuenza	H2: e,n,x	Distinct	9	0.02	ST897-Kimuenza	HC400_24937	Figure S6
48	Panama	H: l,v	Distinct	197	0.43	ST48-Panama	HC400_369	Figure S7
48	Miami	H: a	Distinct	4	0.01	ST48-Miami	HC400_2307, HC400_67476	Figure S7
2019	Napoli or Zaiman	H:z13 or H:v	Mixed	21	0.05	ST2019-Napoli	N/A—interspersed	Figure S8
226	Carrau	O:6,14	Distinct	4	0.01	ST226-Carrau	HC400_363	Figure S9
226	Gatow	O:6,7	Distinct	3	0.01	ST226-Gatow	HC400_59526	Figure S9
684	Uganda or Sinstorf	H:l,v,or H: l,z13	Mixed	75	0.16	ST684-Uganda	N/A—interspersed	Figure S10
909	Bareilly	H: 1,5	Distinct	299	0.65	ST909-Bareilly	HC200_899	Figure S11
909	Richmond	H: 1,2	Distinct	86	0.19	ST909-Richmond	HC200_101	Figure S11
2256	Brunei or tananarive	O: 8, 20 or O: 6, 8	Mixed	7	0.02	ST2256-Brunei	N/A—interspersed	Figure S12
101	Bochum	H: r	Distinct	4	0.01	ST101-Bochum	HC900_491	Figure S13
101	Wien	H: b	Distinct	5	0.01	ST101-Wien	HC900_95	Figure S13

^a^Not used in MAC typing but presented for comparison and explanation.

^b^Any serotype prediction programme can be used though some antigens cannot be distinguished.

^c^Figure shows core SNP cluster for each antigenic type.

^d^Hierarchical Clustering Level at which the serovars can be differentiated genetically.

No. = number of MAC type, % of the 46,268 Salmonella reported.

**Figure 1:**
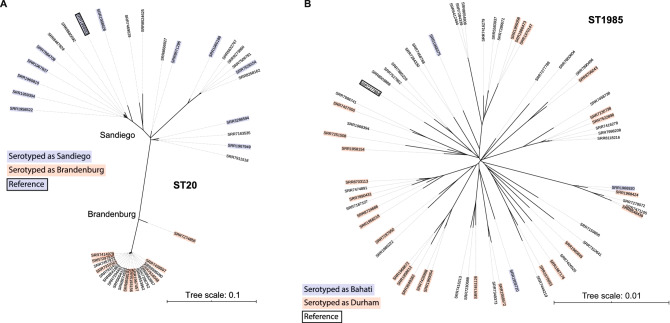
(**A**) Phylogenetic analysis of MAC types Brandenburg and Sandiego (ST20). Representative strains were serotyped and serotype result is highlighted in blue (Sandiego) or red (Brandenburg). (**B**) Phylogenetic analysis of MAC types Bahati and Durham (ST1985). Representative strains were serotyped and serotype result is highlighted in blue (Bahati) or red (Durham).

#### Somatic clusters

There were three STs containing multiple serovars differentiated by O antigens. Comparison between antibody-based serotyping and the SeqSero predictor programmes showed that SeqSero could not predict all antigenic expressions for the somatic antigen. The common antigens involved in the sequence-based naming issues were O22 and O23 (ST1985, Fig. 1B), O6,7 and O6,14 (ST226, see Supplementary Fig. S9) and O6,8 and O8,20 (ST2256, see Supplementary Fig. S1). Phylogenetic clustering showed that within a single ST, two serovars, if differentiated only by O antigen, did not separate into distinct clusters (Table 1). The exception to this was the serovar Carrau or Gatow (ST226, See supplementary Fig. S9).

#### Flagella clusters

There were fourteen STs containing multiple serovars with an H antigen difference (Table 1). Comparison between antibody-based serotyping and the SeqSero predictor programmes showed that SeqSero could predict all antigens from the *fliC* and *fljB* gene sequences. The most common antigens involved in the sequence-based naming issues were the H antigens: *fliC*,: l,v; e,h; z10; z23; z13; a; b; d; r and *fljB*: 1,2; ; 1,5 1,7; z-15; e,n,x. Phylogenetic clustering showed that the majority of serovars found in the same ST, which differed by H antigen, formed distinct clades (ST20-Fig. 1A, ST49-Fig. S2, ST582-Fig. S3, ST22-Fig. S4, ST241-Fig. S5, ST897-Fig. S6, ST48-Fig. S7, ST909-Fig. S11 and ST101-Fig. S13) and so were considered as separate entities (Table 1). There was also an example of a polyphyletic serovar found in two problematic groups defined as MAC type ST241-Bredeney and ST897-Bredeney but still genetically distinct (Table 1, Fig. S14). The exceptions were two groups that contained mixed clades (Table 1, ST2019-Fig. S8 and ST684-Fig. S10) which were differed by the l,v and l,v,z13 antigen (Table 1).

#### Major antigenic cluster (MAC) typing

We then used core SNP clustering to test if the different serovars, defined by traditional serotyping, separated into distinct phylogenetic clusters or were distributed together as a mixed cluster; this we named MAC typing and the groupings were confirmed by cgMLST Hierarchal Clustering. Where the phylogenetic clusters contained mixed antigenic types the most common serovar seen by the laboratory was used to name all isolates in that cluster. Phylogenetic analysis and cgMLST Hierarchal Clustering showed that distinct MAC types varied in their genetic relatedness and split from a range of 100 alleles level (ST22, see Supplementary Fig. S4) to 900 alleles level (ST582, see Supplementary Fig. S3) (Table 1).

### Novel STs

Analysis in this study showed that novel STs (n = 827) make up a small percentage of *Salmonella enterica* subspecies *enterica* but the number of new STs arising continues to be stable accounting for 1.8–2.4% of *Salmonella enterica* subspecies *enterica* reported each year (Table 2). The novel bioinformatic pipeline was not fully implemented in 2014 and novel STs were unrepresented in that year.

**Table 2:** Number of Novel STs identified each year at PHE.

Year	No. novel ST	Total Salmonella reported	% of novel ST to Salmonella reported
2014*	18	6406	0.3*
2015	165	8374	2.0
2016	217	8930	2.4
2017	180	9381	1.9
2018	180	10,143	1.8
2019 (Jan–June)	67	3034	2.2
Total	827	46,268	1.8

*Automated Novel ST pipeline implemented in 2015 so Novel STs underrepresented in 2014 in this analysis.

In summary, of the 46,268 *Salmonella* analysed, using genomic testing and a polyphasic approach of ST, SeqSero and MAC typing enabled 99.96% of isolates to be reported as a named serovar. For 21 (0.04%) novel ST isolates, only a partial antigenic profile, according to the Kauffman-White Scheme could be characterised (see Supplementary Fig. S1).

## Discussion

Based on the analysis reported here, we have shown that MLST and SeqSero can differentiate the majority of *Salmonella* previously defined isolates into major antigenic clusters—MAC types, which map to serovars in the Kauffmann-White scheme. The assignment of a MAC type to the 0.37% of problematic and novel isolates resulted in the total genomic identification of 99.96% of isolates (see Supplementary Fig. S1). The 0.04% of isolates which could not be fully resolved in terms of the full antigen prediction was still an improvement to 2.1% of isolates that could not be resolved via antibody-based testing which were reported as ‘unnamed *Salmonella*’. Although MAC typing of problematic groups depended initially upon core genome SNP typing, this will not need to be repeated for the STs described here. Assignment of a serovar name to an isolate with a sequence type can be achieved through many programmes freely available online^11,13,26^. Laboratories using WGS can use this publication to ascribe serovar names to the 14 problematic STs described here. This publication also provides a road map for assigning serovar names to any new problematic STs seen in other regions of the world.

There were fourteen polyserovar groups addressed in this paper which contains a comprehensive dataset of *Salmonella* reported in England and Wales, it is likely that there will be more groups in the future and in different regions of the world. Analysis of the groups showed that genoserotyping programmes can usually differentiate polyserovars that differ by the flagella antigen using current software programmes such as SeqSero^11^ and that these polyserovars, were genetically different. Therefore, those serovars which form distinct clades, should continue to be differentiated even though they are in the same ST. There are exceptions to this rule including those differentiated by the lv,lz13 antigen (Serovar Uganda or Sinstorf) or the H:z13 or H:v antigen (Serovar Napoli or Zaiman) which formed mixed clades. This is likely due to the quality or specific binding properties of the antibodies. Interpretation of results may also be a factor as shown by MAC types ST22-Braenderup (H:e,n,z15) and ST22-Larochelle (H:1,2), a clearly distinct group, in which antibody-based results incorrectly identified some of the strains (see Supplementary Fig. S8), (Table 1, Figs. 1A, see Supplementary Figs. S2–S7, S10, S11, S13).

Polyserovars that differed by the somatic antigen could not be differentiated by WGS genoserotyping software programmes and the majority were not genetically different (Table 1, Fig. 1B, see Supplementary Fig. S12). With the exception of the distinct MAC Types such as ST226-Gatow and ST226-Carrau (see Supplementary Fig. S9). Even though genoserotyping cannot differentiate these groups, Hierarchical Clustering can be used, in this case at the 400-allele level (Table 1). The exceptions from the majority of strains where differing flagella antigens are genetically distinct and differing somatic antigens are mixed clades is the reason why assessment of each problematic group for MAC typing is initially required. There does not appear to be any biological relevance to differentiating groups with mixed clades and we recommend that the most common name is used for mixed clade groups while we wait for international consensus. Using the most common MAC type will not be consistent across all countries but the use of public databases, such as Enterobase, may assist in deciding the most common international name. Ultimately, it is important for the classification scheme to be updated to ensure global consistency of nomenclature. Our recommendation is to withdraw (such as *S.* Bahati) or reinstate historical serovar names (such as *S.* Java) as defined by the Kauffmann-White scheme based on an ST-serovar convention.

If we are to replace serology with sequencing entirely then the current approach of validating novel STs via serotyping needs to change. The analysis in this study showed that novel STs (n = 827) made up 1.78% of *Salmonella* referred (see Supplementary Fig. S1) and that this percentage was stable over 4 years (Table 2). Our data suggests the possibility of an open population with a fairly consistent number of novel STs emerging each year (Table 2) or a massive population size of *Salmonella* world-wide. Either way, we have not reached a plateau for the discovery of new STs and so we need a way to name them. Currently PHE validate all novel STs phenotypically when only 0.04% can’t be genotypically predicted due to issues with antigen prediction software. *Salmonella* has a complex system for expression of antigens and the current publicly available prediction software, SISTR^13^ and SeqSero^11^ are not able to predict all somatic antigens due to the way somatic antigens are encoded and expressed. There are also issues where predictions will not always relate to phenotypic expression^12,27^. This may be due to mutations in the gene or non-specificity of the antigens as rearrangements and mutations cannot be easily predicted by gene detection in software programmes and discrepancy between genotype and phenotype may occur. Historical methods for naming new serotypes will also play a role, if the antigen had historically reacted with a known antibody, even if non-specific, it was recorded as being the same. We see evidence of this when looking at sequences of the flagella from 6,7:c:1,5 strains (*S.* Decatur and the *S.* Choleraesuis/*S.* Paratyphi C group, originally differentiated by biotyping) in which antibody-based testing is not efficient as distinguishing distinct sequences of *fljB* genes which has resulted in serological conflation of these genetically unrelated serovars^9^. We see the same issue where genoserotyping can’t differentiate historical biotyped groups with *S.* Paratyphi B and *S.* Paratyphi B *var* Java, which can’t be differentiated serologically. They are genetically distinct with the former causing invasive disease^28^ and fall into distinct ST groups^9^ and therefore should be clearly differentiated in name. The use of MAC typing could also resolve these groups without the need for biochemical testing to differentiate biotypes. Essentially, SISTR and SeqSero databases are based on the K&W scheme which depends upon the excellent but slightly flawed serotyping.

Currently for novel *Salmonella*, antibody-based serology is still undertaken to comply with the Kauffmann-White scheme which does not take the genotype into account. The impact of continuing to use antibody based serological methods includes: the continued use of animal model products; increase in turnaround times by 3–14 days; additional staff resources and expertise; and additional quality testing systems and cost. Serology still holds value in microbiology, retaining the skills in specific institutes (as with viral culture) will be important for the future. It is also useful for frontline laboratories where presumptive identification is required and in low to middle income countries who don’t have access to molecular techniques. At the very least however, the concept of using ST, genotypic expression and MAC typing for defining *Salmonella* where genomic methods are available should be adopted. Particularly in reference laboratories and accepting that 0.04% may not predict all phenotypic expressions but that there is a very low impact in clinical or public health management. It is unlikely that a full reform of the Kauffmann and White naming scheme based on genetic differences will occur, to differentiate polyphyletic or polyserovar groups but the use of MAC types will resolve *Salmonella* nomenclature issues.

This study/opinion piece supports the continued use of historical names, they are valuable in terms of international communication and the understanding of biological, clinical, transmission and outbreak association of groups. It is recommended that *Salmonella* are named genetically as the gold standard where these practices are available. To define new MAC types, either SNP or cgMLST hierarchical clustering approaches, with a minimum of three of each serovar, could be used as this study shows. Although these methods can be performed via bespoke bioinformatic platforms, both methodologies are also available on Enterobase and don’t require extensive bioinformatic skills. Having a strict cut off to define MAC types is not possible because the genetic variability within different groups of Salmonella varied between HC100-HC900 for defining MAC types (Table 1). Therefore, these distinctions would be required on a group by group basis.

### Definition of Salmonella in the genomic era

The MAC type can be inferred by validated genomic approaches with validated databases, for example by ST^9,10^, prediction software programmes^11,13,27^ and MAC typing (this study). It should be recognised that for novel STs, not all somatic antigens will be predicted and so an agreed approach to naming is required. It is therefore recommended that *Salmonella* strains are named first by *Salmonella* species and subspecies and then the MAC type (ST or provisional ST plus historical serovar name or Serogene). Examples of MAC types: (a) *S. enterica* ST34—*S.* Typhimurium-monophasic variant (b) *S. enterica* ST43—*S.* Java (c) *S*. *enterica* ST86—*S.* Paratyphi B (d) *S*. *diarizonae* ST1262—18:l,v:z, (e) *S. bongori*, ST398—60:z41:-.

## Conclusion

A previous report from PHE, using the same dataset, stated that almost half of sequenced *Salmonella* isolates that were checked phenotypically were due to discrepancies with the findings from the serology of sending laboratory^14^. We show here that this is not necessary as the serovar designation from genome sequence data alone can be trusted and used for reporting. Using WGS, 99.96% of *Salmonella* isolates reported in the UK can currently be assigned a MAC type name taken from the MLST^9,16^ and genetic antigenic profile in line with the Kauffman and White Scheme ^1,3^. We recommend a change to using sequence data for the routine naming of all *Salmonella*.

## Supplementary Information

Supplementary Legends.

Supplementary Figure S1. Overview of genomic methods for Salmonella serovar identification

Supplementary Figure S2. Phylogenetic analysis of MAC types ST49 - S. Hafia and ST49 - S. Saintpaul

Supplementary Figure S3. Phylogenetic analysis of MAC types ST582 - S. Chailey and ST582 - S. Kottbus

Supplementary Figure S4. Phylogenetic analysis of MAC type ST22 - S. Braenderup and ST22 - S. Larochelle

Supplementary Figure S5. Phylogenetic analysis of MAC types ST214 - S. Bredeney and ST214 - S. Schwarzengrund

Supplementary Figure S6. Phylogentic analysis of MAC types ST897 - S. Bredeney and ST897 - S. Kimuenza

Supplementary Figure S7. Phylogenetic analysis of MAC types ST48 - S. Panama and ST48 - S. Miami

Supplementary Figure S8. Phylogenetic analysis of MAC type ST2019 - S. Napoli (with serovar Zaiman)

Supplementary Figure S9. Phylogenetic analysis of MAC types ST226 - S. Carrau and ST226 - S. Gatow

Supplementary Figure S10. Phylogenetic analysis of MAC types ST684 - S. Uganda (with serovar Sinstorf)

Supplementary Figure S11. Phylogenetic analysis of MAC types ST909 - S. Bareilly and ST909 - S. Richmond

Supplementary Figure S12. Phylogenetic analysis of MAC type ST2256 - S. Brunei (with serovar Tananarive)

Supplementary Figure S13. Phylogenetic analysis of MAC types ST101 - S. Bochm and ST101 - S. Wein

Supplementary Figure S14. Phylogenetic analysis of MAC types ST241 S. Bredeney and ST897 - S. Bredeney
