# Supplementary material for: Salmonella nomenclature in the genomic era: a time for change
Source: Sci Rep. 2021 Apr 5;11:7494. doi: 10.1038/s41598-021-86243-w (PMC8021552; doi:10.1038/s41598-021-86243-w)
Supplement: Supplementary file 1 — Supplementary Legends. [file 41598_2021_86243_MOESM1_ESM.docx]

# *Salmonella* Nomenclature in the Genomic Era - a Time for Change

Marie Chattaway^1^*, Gemma C. Langridge^2^ and John Wain^2,3^

^1^ Gastrointestinal Bacteria Reference Unit, Public Health England, London, UK, NW9 5EQ:

^2^ Quadram Institute Bioscience, Norwich Research Park, UK, NR4 7UQ,

^3^ Norwich Medical; School, University of East Anglia

*Corresponding Author

Marie Anne Chattaway

Gastrointestinal Bacteria Reference Unit

*Salmonella* Reference Service

Public Health England

London, UK

Email: [marie.chattaway@phe.gov.uk](mailto:marie.chattaway@phe.gov.uk)

**Supplementary Figures**

**Figure S1** - Overview of genomic methods for *Salmonella* serovar identification

**Figure S2** – Phylogenetic analysis of MAC types ST49-Haifa and ST49-Saintpaul

**Figure S3** - Phylogenetic analysis of MAC types ST582-Chailey and ST582-Kottbus

**Figure S4** - Phylogenetic analysis of MAC types ST22-Braenderup and ST22-Larochelle

**Figure S5** - Phylogenetic analysis of MAC types ST241-Bredeney and ST241-Schwarzengrund

**Figure S6** - Phylogenetic analysis of MAC types ST897-Bredeney and ST897-Kimuenza

**Figure S7** - Phylogenetic analysis of MAC types ST48-Panama and ST48-Miami

**Figure S8** - Phylogenetic analysis of MAC type ST2019-Napoli (with serovar Zaiman)

**Figure S9** - Phylogenetic analysis of MAC types ST226-Carrau and ST226-Gatow

**Figure S10** - Phylogenetic analysis of MAC type ST684-Uganda (with serovar Sinstorf)

**Figure S11** - Phylogenetic analysis of MAC types ST909-Bareilly and ST909-Richmond

**Figure S12** - Phylogenetic analysis of MAC type ST2256-Brunei (with serovar Tananarive)

**Figure S13** - Phylogenetic analysis of MAC types ST101-Bochum and ST101-Wien

**Figure S14** - Phylogenetic analysis of MAC types ST241-Bredeney and ST897-Bredeney
