# Supplementary figures and images for: Salmonella nomenclature in the genomic era: a time for change
Source: Sci Rep. 2021 Apr 5;11:7494. doi: 10.1038/s41598-021-86243-w (PMC8021552; doi:10.1038/s41598-021-86243-w)

Overview of genomic methods for Salmonella serovar identification  
January 2014 – June 2019

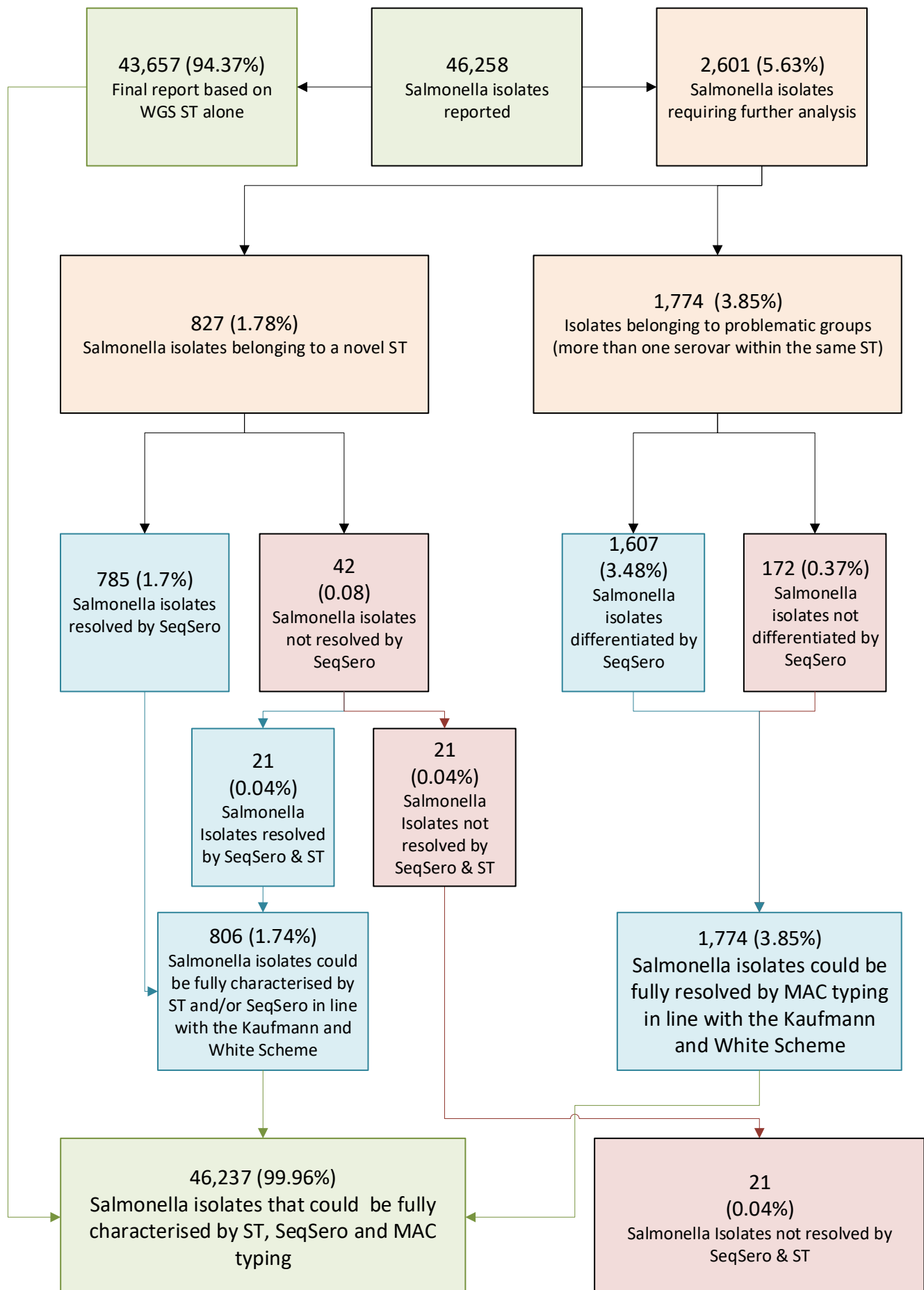

Supplement: Supplementary file 2 — Supplementary Figure S1. Overview of genomic methods for Salmonella serovar identification [file 41598_2021_86243_MOESM2_ESM.pdf]

# ST49

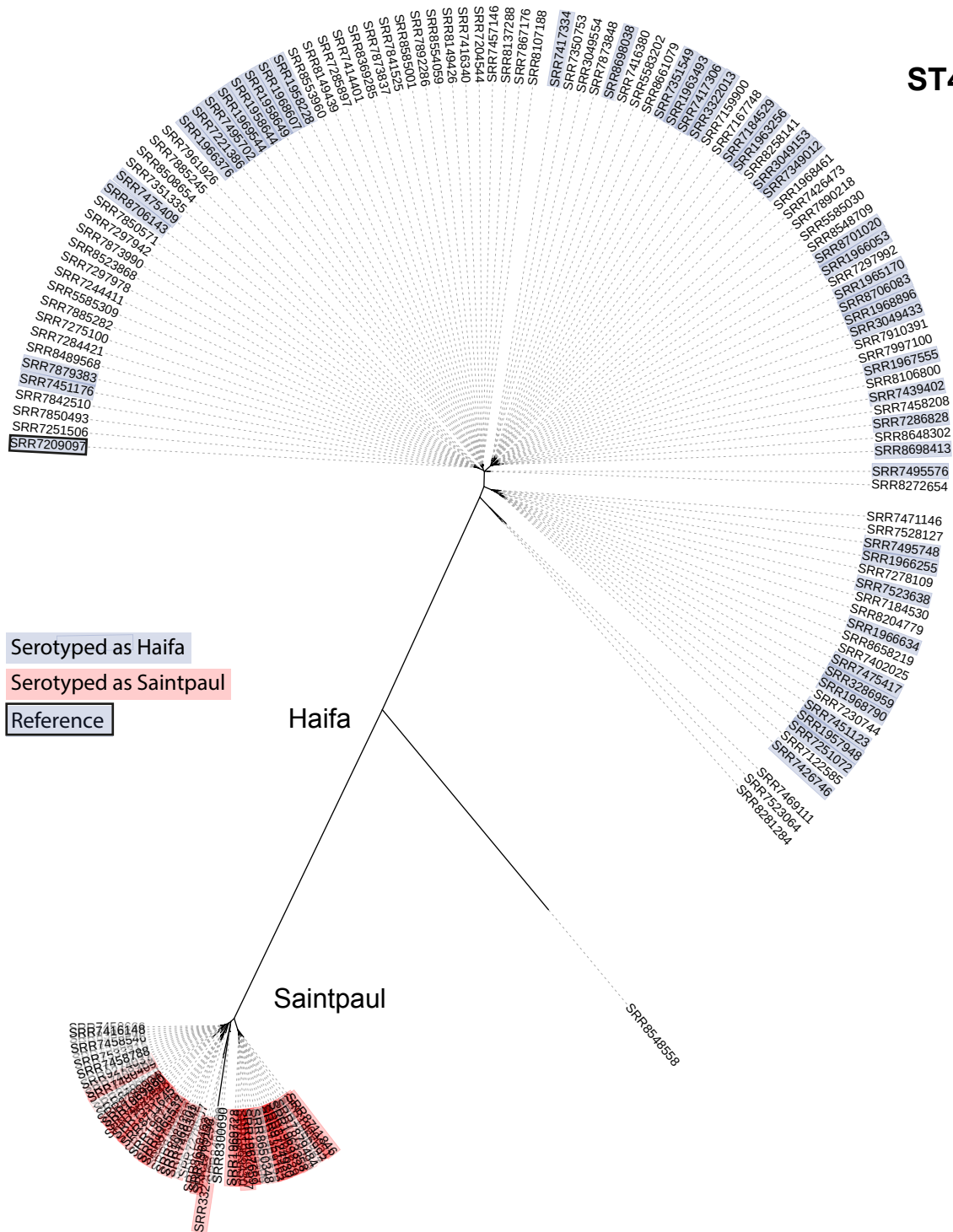

Tree scale: 0.01

Supplement: Supplementary file 3 — Supplementary Figure S2. Phylogenetic analysis of MAC types ST49 - S. Hafia and ST49 - S. Saintpaul [file 41598_2021_86243_MOESM3_ESM.pdf]

ST22

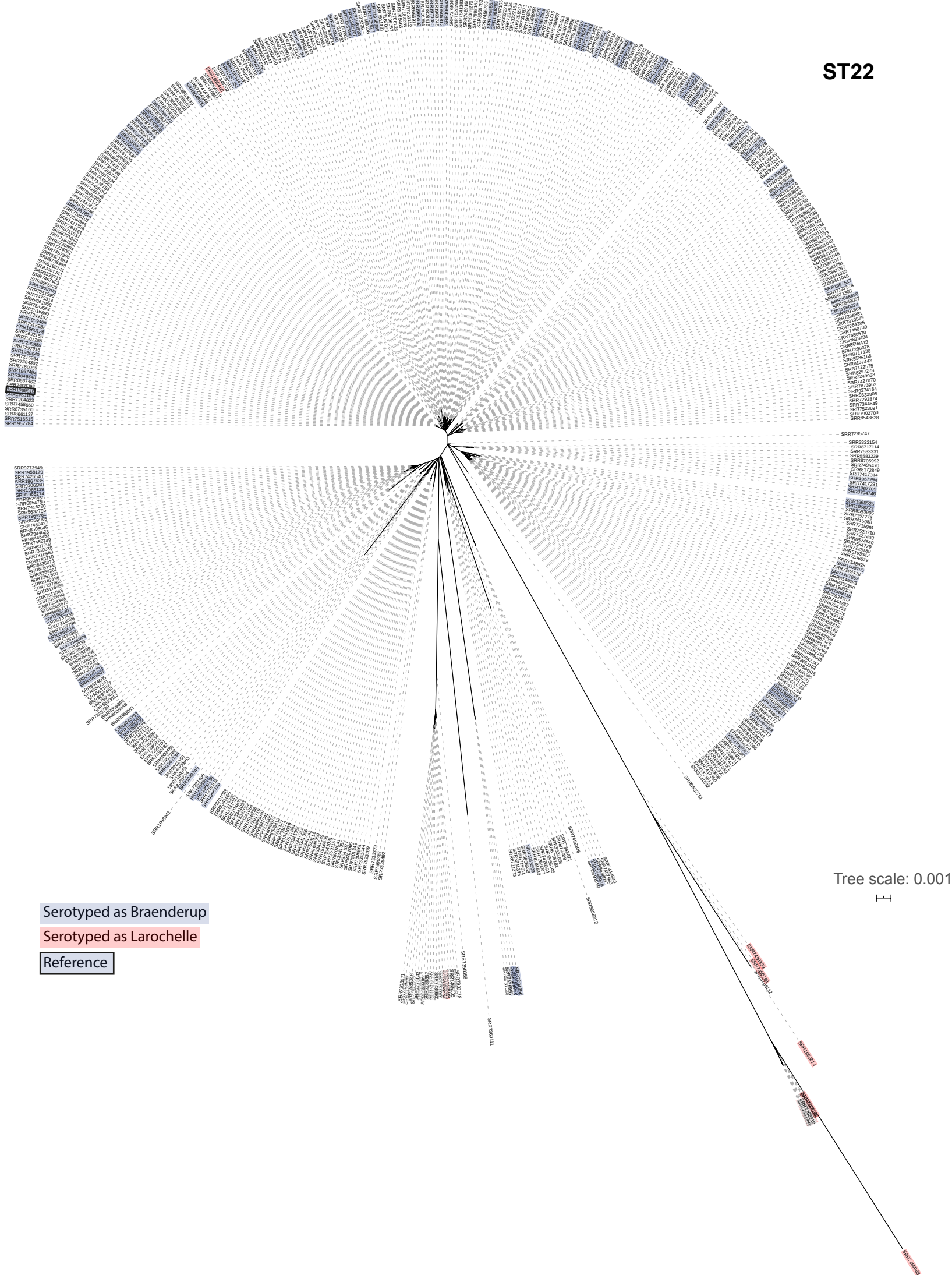

Supplement: Supplementary file 5 — Supplementary Figure S4. Phylogenetic analysis of MAC type ST22 - S. Braenderup and ST22 - S. Larochelle [file 41598_2021_86243_MOESM5_ESM.pdf]

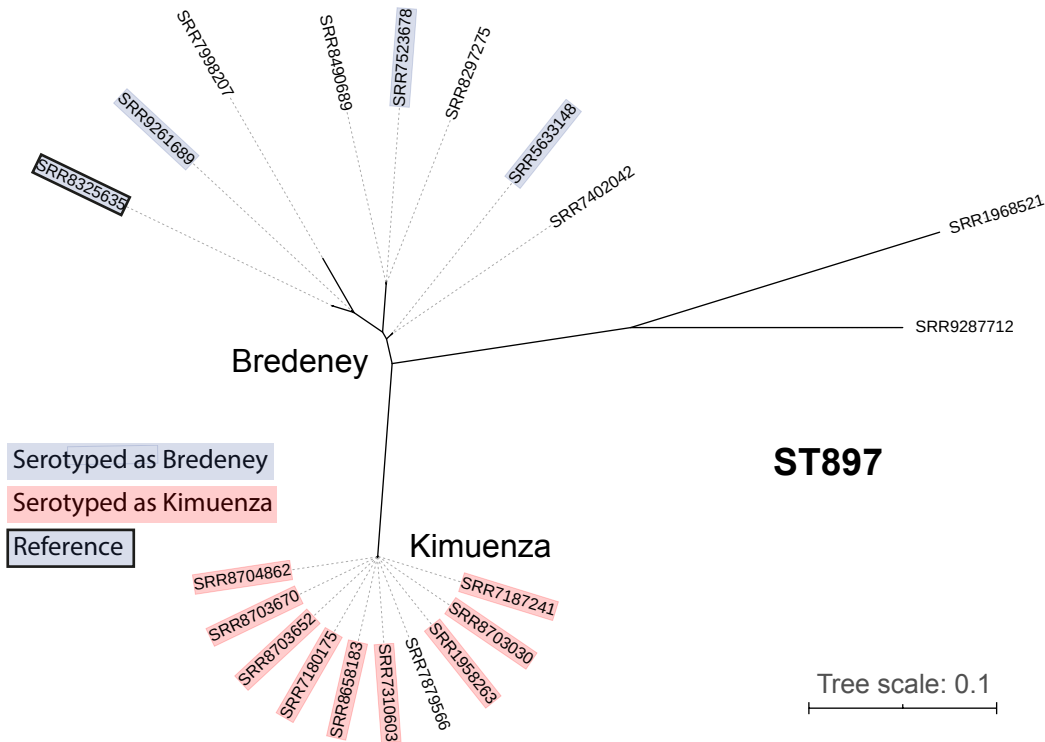

Supplement: Supplementary file 7 — Supplementary Figure S6. Phylogentic analysis of MAC types ST897 - S. Bredeney and ST897 - S. Kimuenza [file 41598_2021_86243_MOESM7_ESM.pdf]

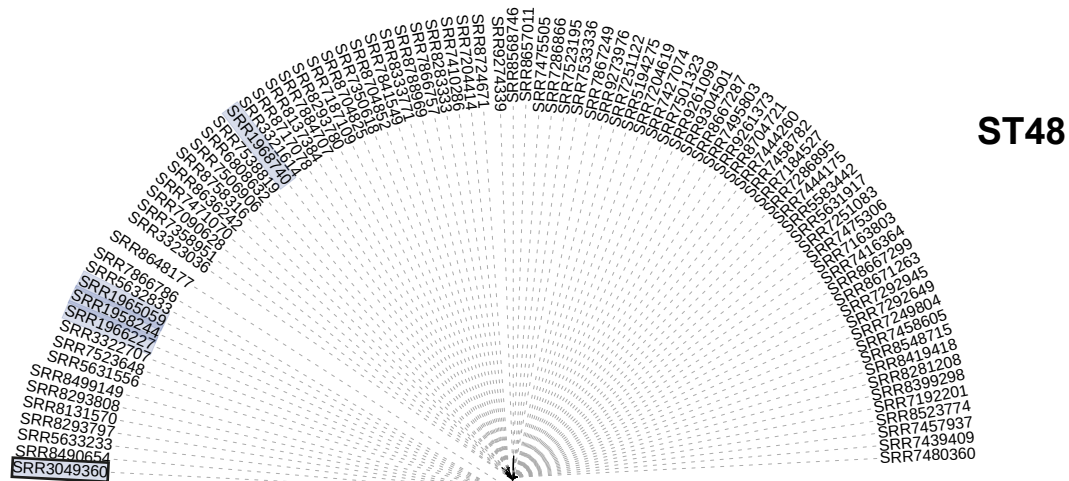

ST48

Panama

Miami

Tree scale: 0.01

Serotyped as Panama

Serotyped as Miami

Reference

SRR8711747  
SRR7221422

Supplement: Supplementary file 8 — Supplementary Figure S7. Phylogenetic analysis of MAC types ST48 - S. Panama and ST48 - S. Miami [file 41598_2021_86243_MOESM8_ESM.pdf]

Serotyped as Zaiman  
Serotyped as Napoli  
Reference

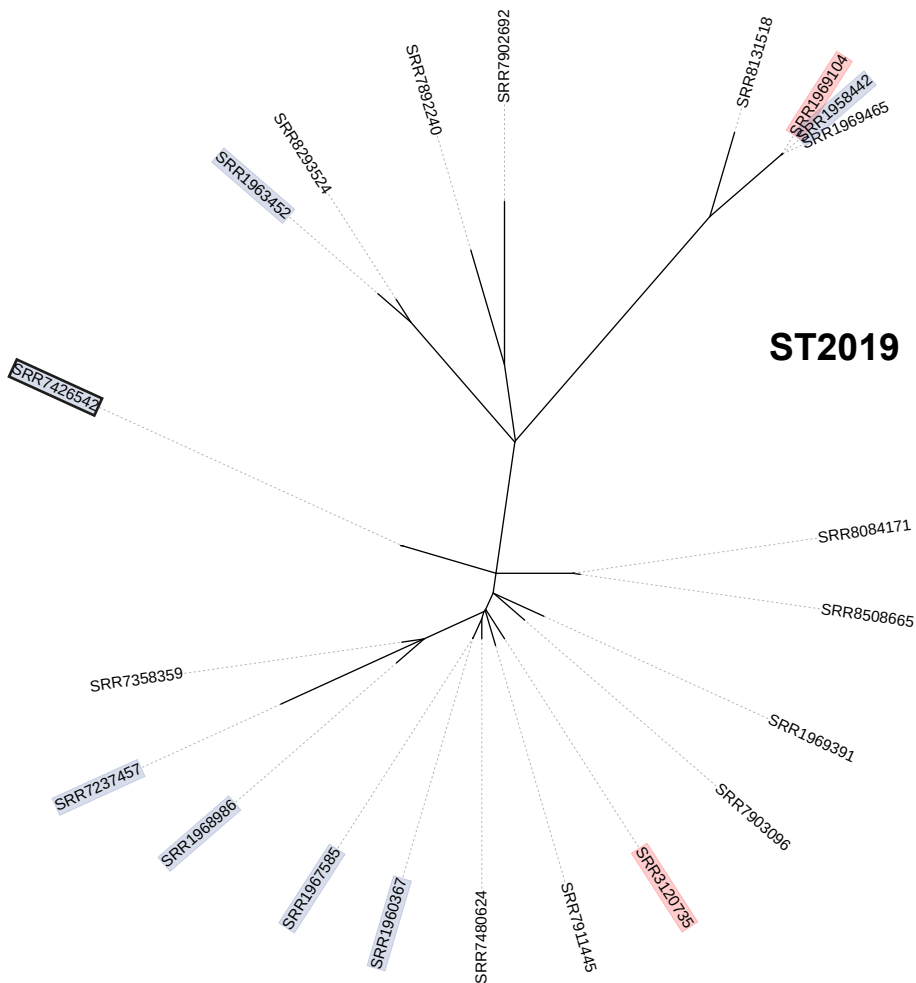

Tree scale: 0.1

Supplement: Supplementary file 9 — Supplementary Figure S8. Phylogenetic analysis of MAC type ST2019 - S. Napoli (with serovar Zaiman) [file 41598_2021_86243_MOESM9_ESM.pdf]

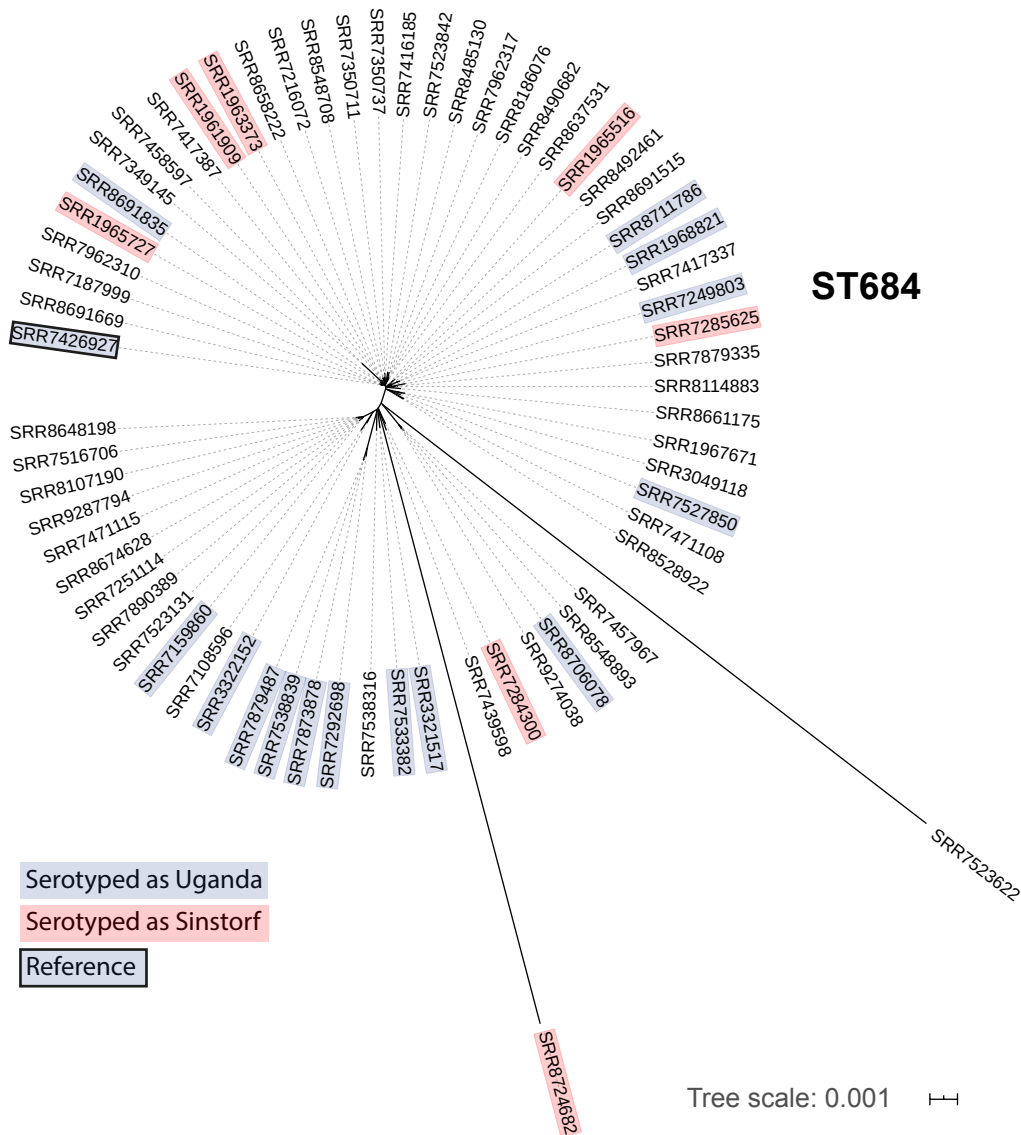

Supplement: Supplementary file 11 — Supplementary Figure S10. Phylogenetic analysis of MAC types ST684 - S. Uganda (with serovar Sinstorf) [file 41598_2021_86243_MOESM11_ESM.pdf]

ST909

Bareilly

Richmond

Serotyped as Bareilly

Serotyped as Richmond

Reference

Tree scale: 0.1

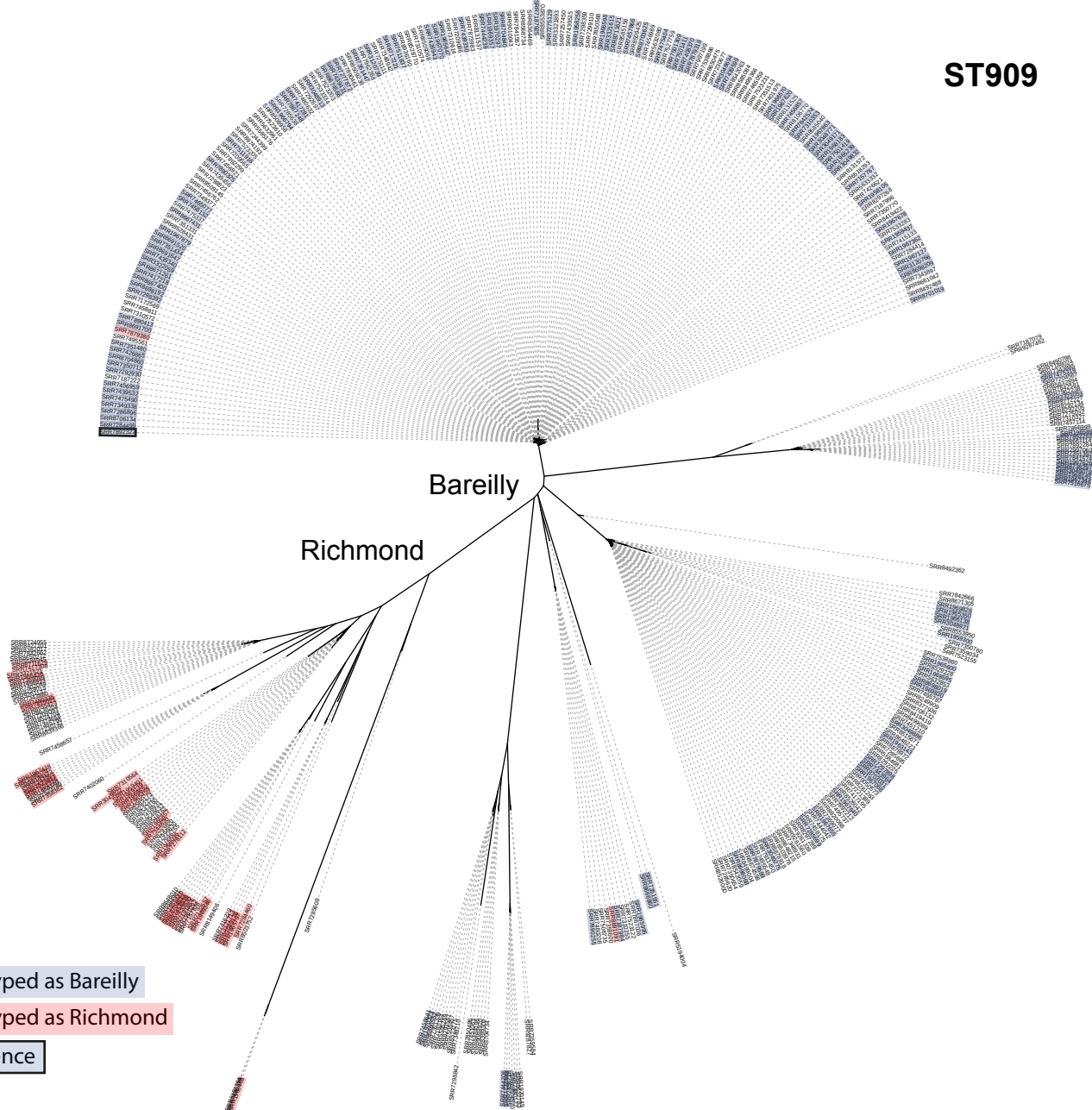

Supplement: Supplementary file 12 — Supplementary Figure S11. Phylogenetic analysis of MAC types ST909 - S. Bareilly and ST909 - S. Richmond [file 41598_2021_86243_MOESM12_ESM.pdf]

# ST2256

Serotyped as Tananarive

Serotyped as Brunei

Reference

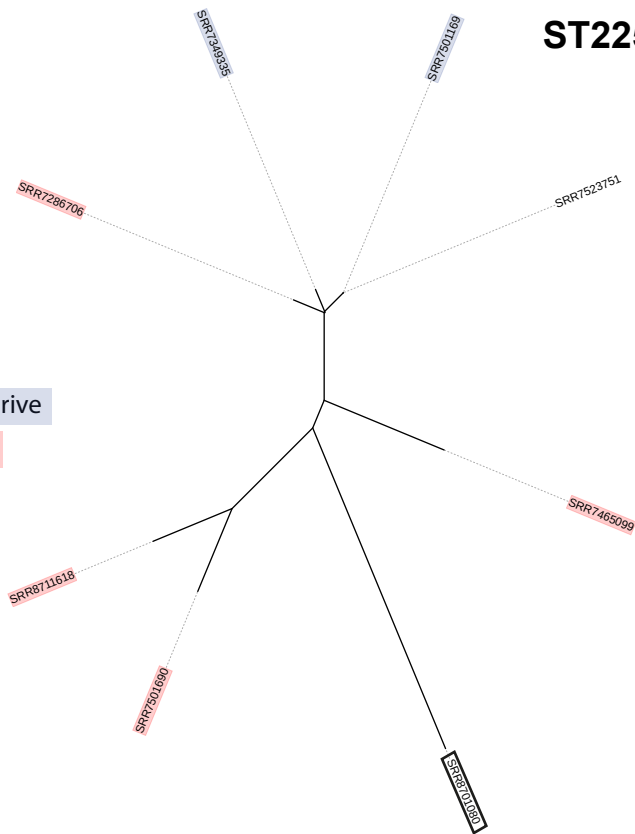

Tree scale: 0.1

Supplement: Supplementary file 13 — Supplementary Figure S12. Phylogenetic analysis of MAC type ST2256 - S. Brunei (with serovar Tananarive) [file 41598_2021_86243_MOESM13_ESM.pdf]

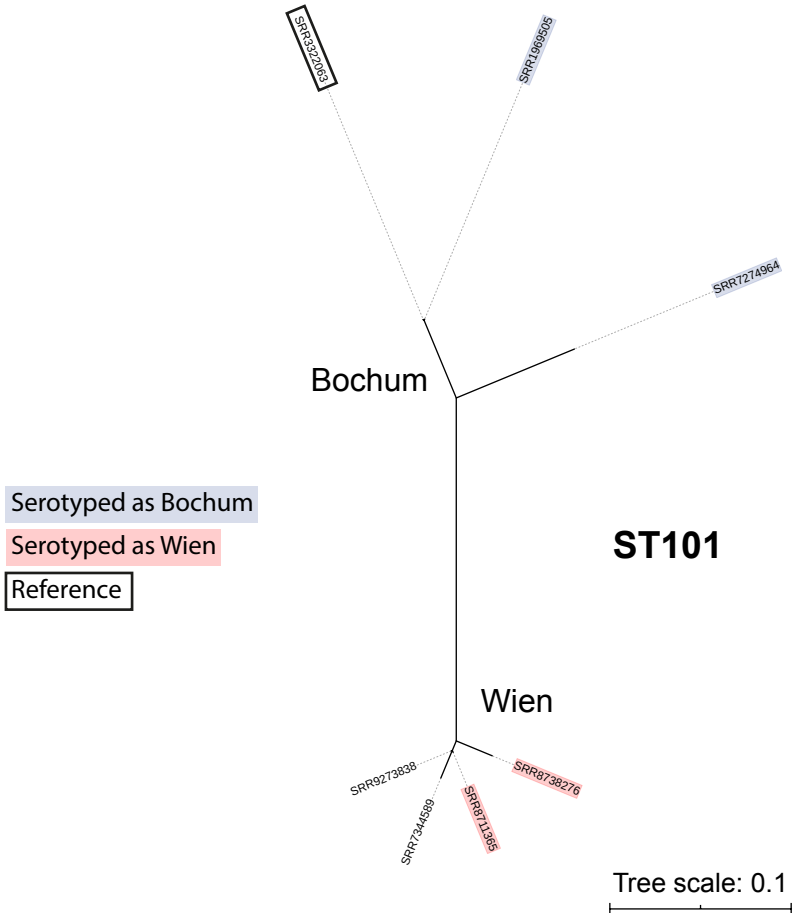

Supplement: Supplementary file 14 — Supplementary Figure S13. Phylogenetic analysis of MAC types ST101 - S. Bochm and ST101 - S. Wein [file 41598_2021_86243_MOESM14_ESM.pdf]

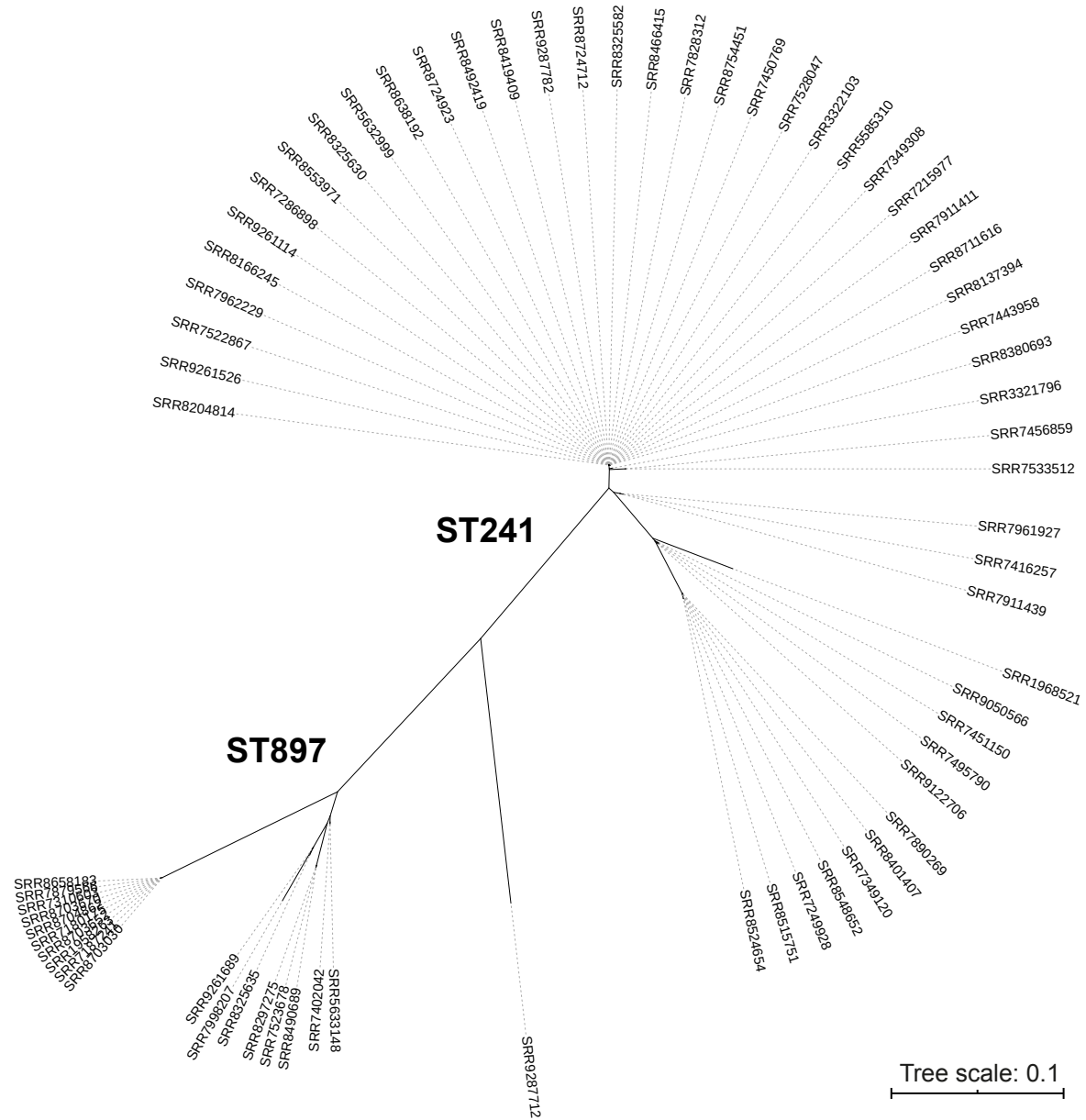

Supplement: Supplementary file 15 — Supplementary Figure S14. Phylogenetic analysis of MAC types ST241 S. Bredeney and ST897 - S. Bredeney [file 41598_2021_86243_MOESM15_ESM.pdf]
