# Supplementary material for: Salmonella nomenclature in the genomic era: a time for change
Source: Sci Rep. 2021 Apr 5;11:7494. doi: 10.1038/s41598-021-86243-w (PMC8021552; doi:10.1038/s41598-021-86243-w)
Supplement: Supplementary file 4 — Supplementary Figure S3. Phylogenetic analysis of MAC types ST582 - S. Chailey and ST582 - S. Kottbus [file 41598_2021_86243_MOESM4_ESM.pdf]

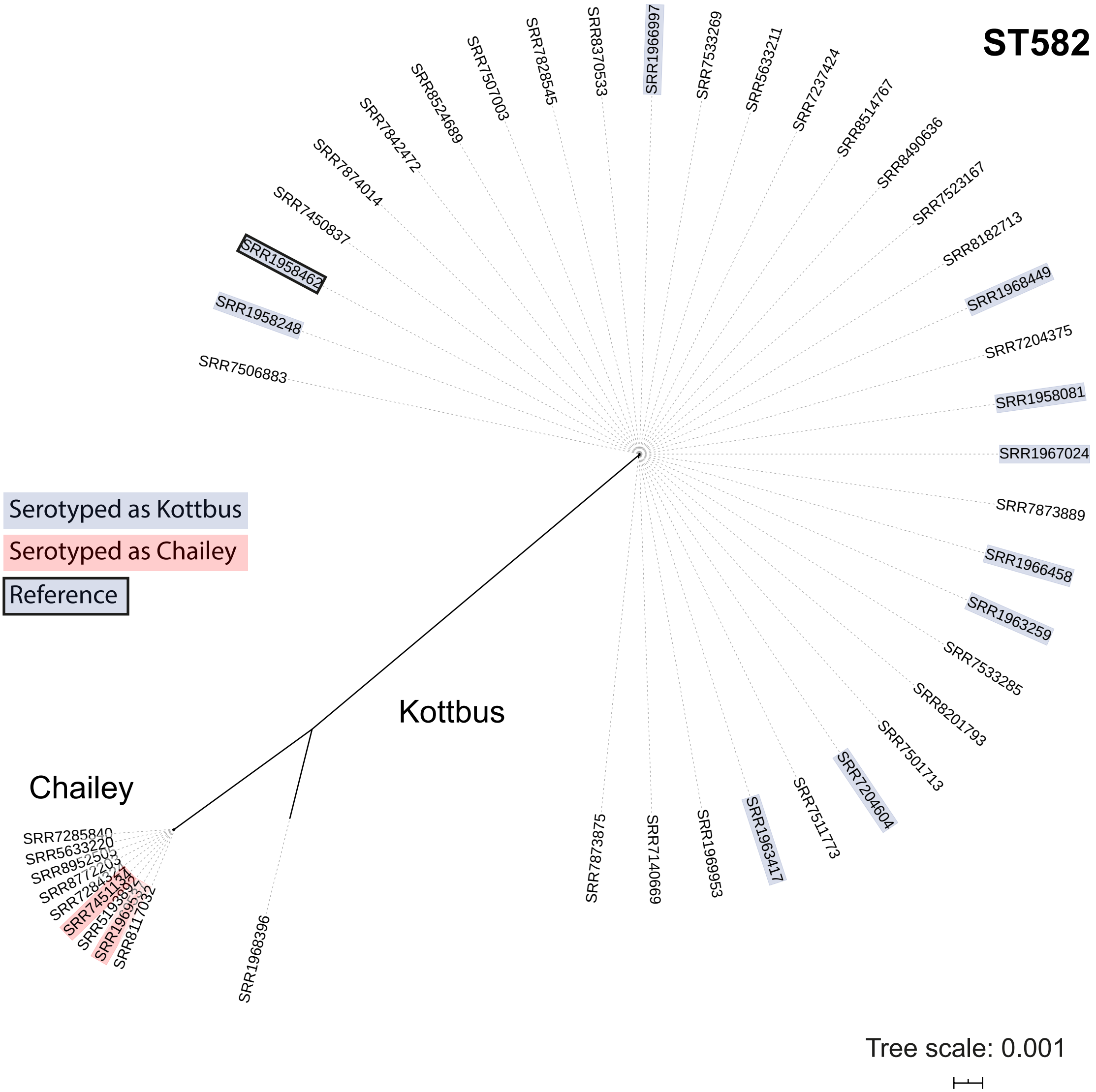

ST582
Serotyped as Kottbus
Serotyped as Chailey
Reference
Kottbus
Chailey
SRR1966997
SRR7533269
SRR5633211
SRR7237424
SRR8514767
SRR8490636
SRR7523167
SRR8182713
SRR1968449
SRR7204375
SRR1958081
SRR1967024
SRR7873889
SRR1966458
SRR1963259
SRR7533285
SRR8201793
SRR7501713
SRR7204604
SRR7511773
SRR1963417
SRR1969953
SRR7140669
SRR7873875
SRR8370533
SRR7828545
SRR7507003
SRR8524689
SRR7842472
SRR7874014
SRR7450837
SRR1958462
SRR1958248
SRR7506883
SRR1968396
SRR7285840
SRR5633220
SRR8952505
SRR8772203
SRR8117032
Tree scale: 0.001
