# Supplementary material for: Salmonella nomenclature in the genomic era: a time for change
Source: Sci Rep. 2021 Apr 5;11:7494. doi: 10.1038/s41598-021-86243-w (PMC8021552; doi:10.1038/s41598-021-86243-w)
Supplement: Supplementary file 6 — Supplementary Figure S5. Phylogenetic analysis of MAC types ST214 - S. Bredeney and ST214 - S. Schwarzengrund [file 41598_2021_86243_MOESM6_ESM.pdf]

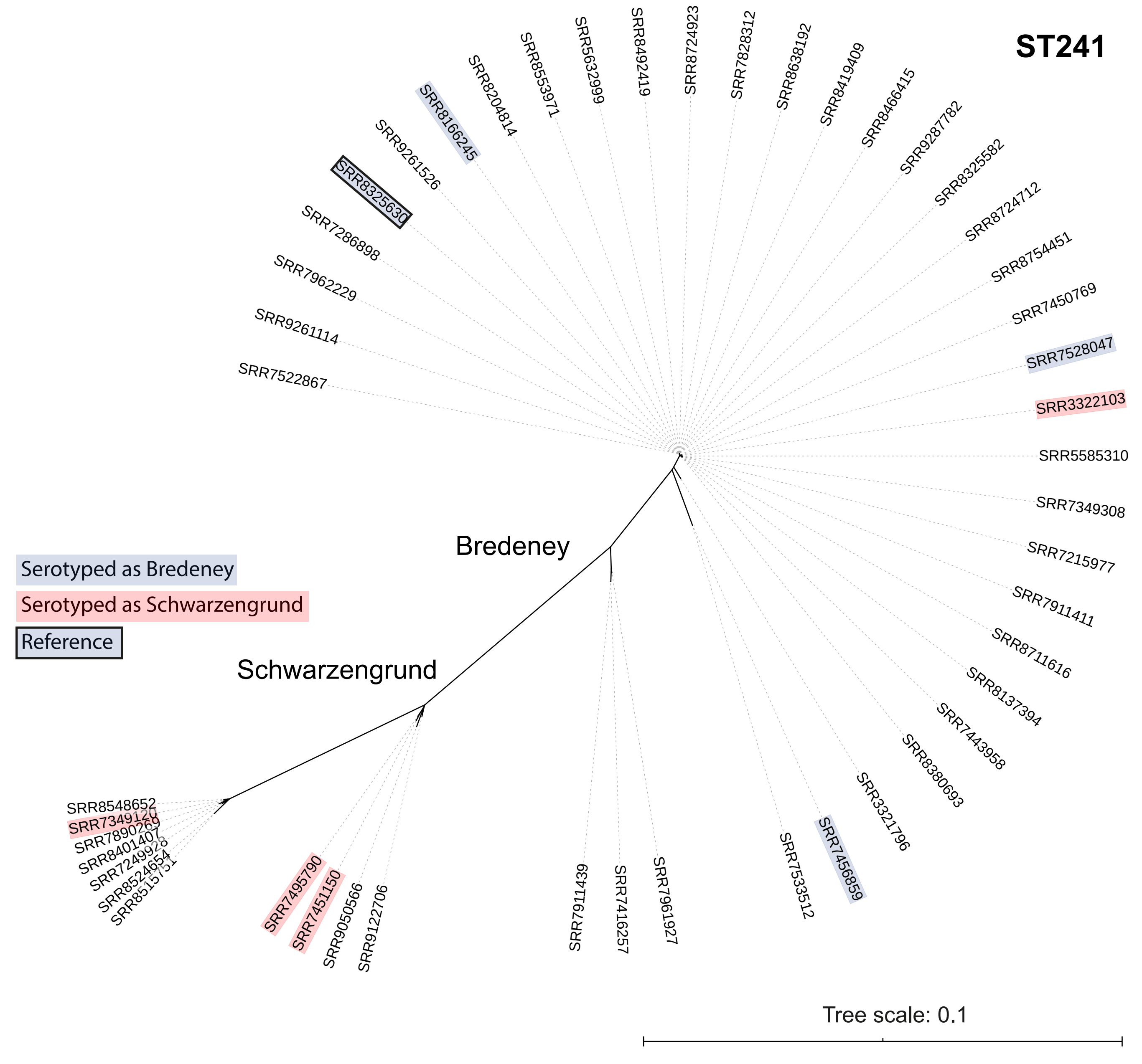
ST241
Bredeney
Schwarzengrund
Serotyped as Bredeney
Serotyped as Schwarzengrund
Reference
Tree scale: 0.1
SRR7522867
SRR9261114
SRR7962229
SRR7286898
SRR8325630
SRR9261526
SRR8166245
SRR8204814
SRR8553971
SRR5632999
SRR8492419
SRR8724923
SRR7828312
SRR8638192
SRR8419409
SRR8466415
SRR9287782
SRR8325582
SRR8724712
SRR8754451
SRR7450769
SRR7528047
SRR3322103
SRR5585310
SRR7349308
SRR7215977
SRR7911411
SRR8711616
SRR8137394
SRR7443958
SRR8380693
SRR3321796
SRR7456859
SRR7533512
SRR7961927
SRR7416257
SRR7911439
SRR9122706
SRR9050566
SRR7451150
SRR7495790
SRR8548652
SRR7349120
SRR7890269
SRR8401407
SRR7249928
SRR8524654
SRR8515751
