# Supplementary material for: Salmonella nomenclature in the genomic era: a time for change
Source: Sci Rep. 2021 Apr 5;11:7494. doi: 10.1038/s41598-021-86243-w (PMC8021552; doi:10.1038/s41598-021-86243-w)
Supplement: Supplementary file 10 — Supplementary Figure S9. Phylogenetic analysis of MAC types ST226 - S. Carrau and ST226 - S. Gatow [file 41598_2021_86243_MOESM10_ESM.pdf]

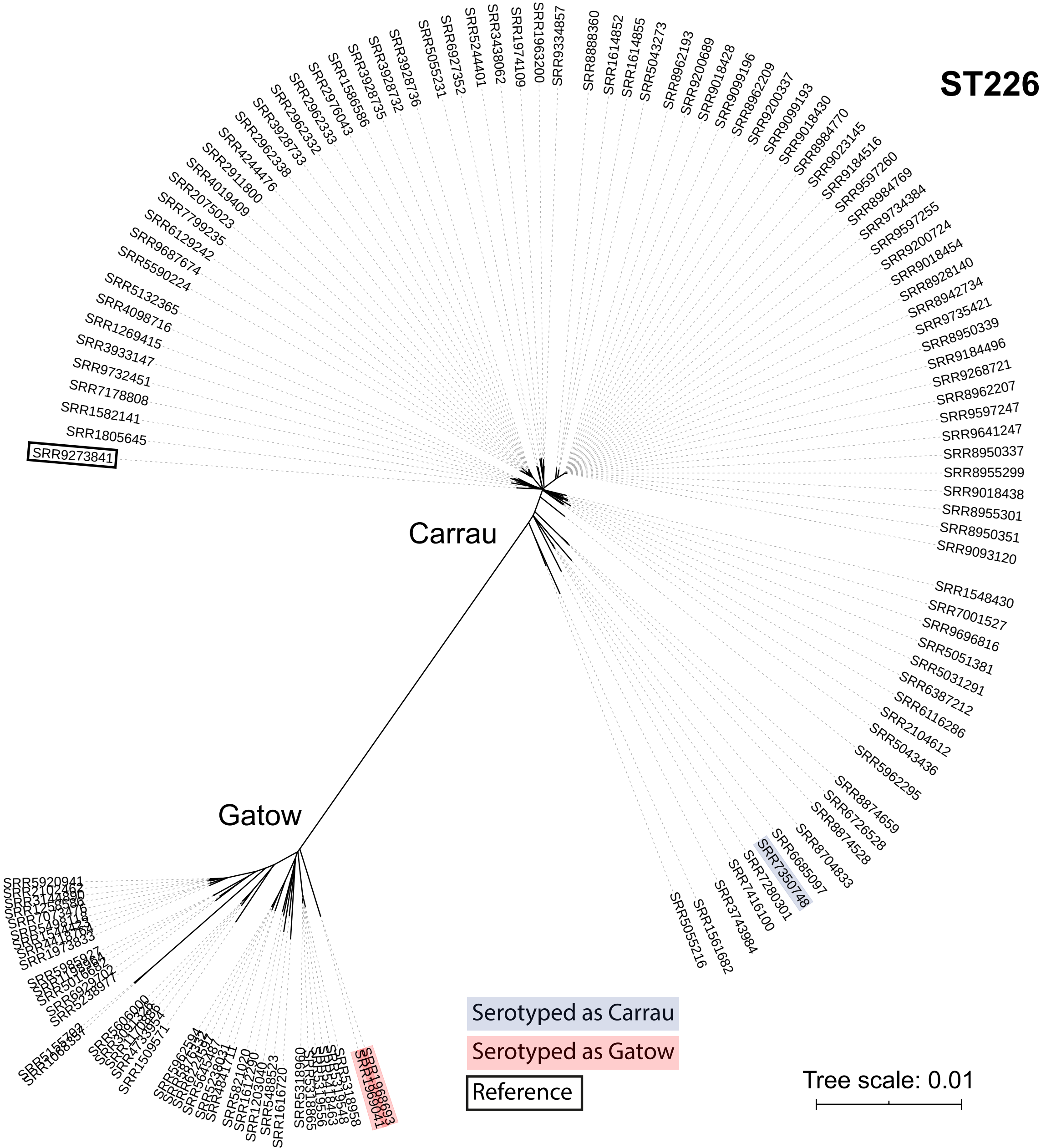
ST226
Carrau
Gatow
SRR9273841
SRR1805645
SRR1582141
SRR7178808
SRR9732451
SRR3933147
SRR1269415
SRR4098716
SRR5132365
SRR5590224
SRR9687674
SRR6129242
SRR7799235
SRR2075023
SRR4019409
SRR2911800
SRR4244476
SRR2962338
SRR3928733
SRR2962332
SRR2962333
SRR2976043
SRR1586586
SRR3928735
SRR3928732
SRR3928736
SRR5055231
SRR6927352
SRR5244401
SRR3438062
SRR1974109
SRR1963200
SRR9334857
SRR8888360
SRR1614852
SRR1614855
SRR5043273
SRR8962193
SRR9200689
SRR9018428
SRR9099196
SRR8962209
SRR9200337
SRR9099193
SRR9018430
SRR8984770
SRR9023145
SRR9184516
SRR9597260
SRR8984769
SRR9734384
SRR9597255
SRR9200724
SRR9018454
SRR8928140
SRR8942734
SRR9735421
SRR8950339
SRR9184496
SRR9268721
SRR8962207
SRR9597247
SRR9641247
SRR8950337
SRR8955299
SRR9018438
SRR8955301
SRR8950351
SRR9093120
SRR1548430
SRR7001527
SRR9696816
SRR5051381
SRR5031291
SRR6387212
SRR6116286
SRR2104612
SRR5043436
SRR5962295
SRR8874659
SRR6726528
SRR8874528
SRR8704833
SRR6685097
SRR7350748
SRR7280301
SRR7416100
SRR3743984
SRR1561682
SRR5055216
SRR5920941
SRR1973833
SRR5238977
SRR1509571
SRR5488523
SRR1616720
SRR5318960
SRR5318865
SRR5318958
Serotyped as Carrau
Serotyped as Gatow
Reference
Tree scale: 0.01
